# CD133 Is a Marker of Bioenergetic Stress in Human Glioma

**DOI:** 10.1371/journal.pone.0003655

**Published:** 2008-11-05

**Authors:** Corinne E. Griguer, Claudia R. Oliva, Eric Gobin, Pascale Marcorelles, Dale J. Benos, Jack R. Lancaster, G. Yancey Gillespie

**Affiliations:** 1 Department of Surgery, Division of Neurosurgery, University of Alabama at Birmingham, Birmingham, Alabama, United States of America; 2 Department of Physiology and Biophysics, University of Alabama at Birmingham, Birmingham, Alabama, United States of America; 3 Department of Anesthesiology, University of Alabama at Birmingham, Birmingham, Alabama, United States of America; 4 Center for Free Radical Biology, University of Alabama at Birmingham, Birmingham, Alabama, United States of America; 5 Center for Glial Biology in Medicine, University of Alabama at Birmingham, Birmingham, Alabama, United States of America; 6 Department of Environmental Health Sciences, University of Alabama at Birmingham, Birmingham, Alabama, United States of America; 7 Department of Microbiology, University of Alabama at Birmingham, Birmingham, Alabama, United States of America; 8 CHU Brest, Hôpital Morvan, Pôle de Biologie-Pathologie, Service d'Anatomie Pathologique, Département de Médecine, Brest, France; Istituto Dermopatico dell'Immacolata, Italy

## Abstract

Mitochondria dysfunction and hypoxic microenvironment are hallmarks of cancer cell biology. Recently, many studies have focused on isolation of brain cancer stem cells using CD133 expression. In this study, we investigated whether CD133 expression is regulated by bioenergetic stresses affecting mitochondrial functions in human glioma cells. First, we determined that hypoxia induced a reversible up-regulation of CD133 expression. Second, mitochondrial dysfunction through pharmacological inhibition of the Electron Transport Chain (ETC) produced an up-regulation of CD133 expression that was inversely correlated with changes in mitochondrial membrane potential. Third, generation of stable glioma cells depleted of mitochondrial DNA showed significant and stable increases in CD133 expression. These glioma cells, termed *rho* ^0^ or ρ^0^, are characterized by an exaggerated, uncoupled glycolytic phenotype and by constitutive and stable up-regulation of CD133 through many cell passages. Moreover, these ρ^0^ cells display the ability to form “tumor spheroids” in serumless medium and are positive for CD133 and the neural progenitor cell marker, nestin. Under differentiating conditions, ρ^0^ cells expressed multi-lineage properties. Reversibility of CD133 expression was demonstrated by transfering parental mitochondria to ρ^0^ cells resulting in stable trans-mitochondrial “cybrid” clones. This study provides a novel mechanistic insight about the regulation of CD133 by environmental conditions (hypoxia) and mitochondrial dysfunction (genetic and chemical). Considering these new findings, the concept that CD133 is a marker of brain tumor stem cells may need to be revised.

## Introduction

Mitochondria play a major role in many cellular processes including energy production, apoptosis, and generation of free radical species. We previously demonstrated that glioma cell lines relied on mitochondrial energy for survival [1], [2], [3]. Moreover, mitochondrial dysfunction has been linked to an up-regulation of glycolysis, which in turn promotes tumor progression and invasion in all cancers [4], [5], [6]. Brain tumor stem cells have been recently identified and characterized [7], [8], [9], [10], [11]. In particular, a discovery was made that an important cell marker, CD133, that is expressed in non-malignant neural progenitor cells, is also expressed by brain tumor stem cells [8], [9], [10], [12], [13], [14]. CD133, also known as prominin-1, is a member of a family of glycoproteins composed of five transmembrane domains, two cytoplasmic loops, two glycosylated extracellular domains, and a cytoplasmic C-terminal domain [15], [16], [17]. To date, there are no known ligands, no signaling mechanisms have been identified and the functional significance of CD133 remains unknown. However, its localization in membrane protusions, filopodia and lamellipodia of mouse neuroepithelial stem cells [18] suggests a function in membrane topology [19]. The cellular mechanisms involved in the regulation of CD133 remain unknown.

Several lines of evidence suggest that cancer stem cells could play an important role in the initiation, progression, and recurrence of many types of cancer. Thus, it is important to understand how these cells are regulated in the context of tumor progression and therapy. One avenue is a possible common microenvironment and bioenergetic pathway between cancer cells and human embryonic stem (hES) cells. Subsequent to implantation and vascularization *in vivo*, hES cells are in a hypoxic environment between 1 to 5% of oxygen [20]. Under these conditions, they remain pluripotent. Because hypoxic embryonic cells cannot produce adequate amounts of ATP via mitochondrial oxidative phosphorylation (OxPhos), they rely on anaerobic metabolism to produce ATP to meet their energy requirements [21], just as many cancer cells do [1], [6].

In this study, we tested the hypothesis that mitochondrial status plays a role in the regulation of the CD133 expression in human glioma cell lines. To this end, we used three cellular models: long term exposure to hypoxia, pharmacologic blockers of the ETC and genetic depletion of mitochondrial DNA (ρ^0^). In all three cellular models, we observed that, regardless of the means used to produce a glycolytic bioenergetic phenotype, CD133 is profoundly up-regulated. Moreover, up-regulation of CD133 expression by hypoxia is reversible upon re-oxygenation. Genetic depletion of mitochondrial DNA (ρ^0^ model) increased CD133 cell surface expression in an irreversible manner and yielded a human glioma model in which CD133 is constitutively expressed. Transfer of mitochondria from parental U251 cells to ρ^0^ cells results in transmitochondrial cybrid clones that manifest parental phenotype. These results strongly demonstrate that mtDNA depletion is at least partly responsible for the CD133 expression.

Our findings, in combination with the uncertain role of CD133, suggest that the use of CD133 expression as a marker for brain tumor stem cells should be critically evaluated.

## Results

### Expression of CD133 increases in glioma cells maintained under hypoxia

**Figure 1:**
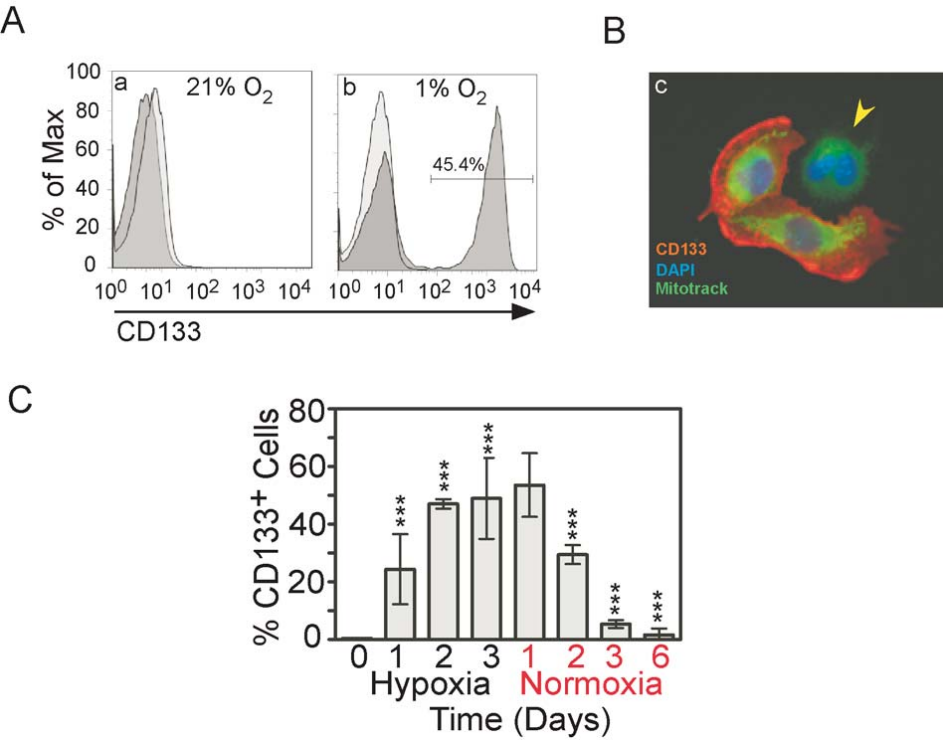
Expression of CD133 in U251 glioma cells under hypoxia. A) Flow cytometry histogram of representative U251 cells, with the first peak representing cells negative for CD133-phycoerythrin expression, and the second peak representing CD133 positive cells. Histogram of U251 in 21% oxygen (a) compared with 1% oxygen (b). Data are representative of five similar experiments. B) Immunofluorescence photographs of representative U251 cells grown in 1% oxygen and processed with CD133 antibody (red). Cytoplasm and nucleus were stained with (1 μM) MitoTracker® (green) and DAPI (blue) respectively. Arrowhead showing one CD133-negative cell in the field. C) Time-dependent expression of CD133 in glioma cells under hypoxia and reoxygenation. Results are given as means ± SD of 5 separate experiments. *** represent p<0.001.

To assess the effect of hypoxia on expression of CD133, we cultured U251MG human glioma cells in 1% oxygen for up to 3 days and compared them to cells maintained under standard normoxic conditions (21% oxygen). Flow cytometric analysis indicated that CD133 expression was not detectable above background when these cells were exposed to 21% oxygen. However, U251MG glioma cells cultured in 1% oxygen were enriched in CD133^+^ cells (Fig. 1A). Using immunostaining, we confirmed that CD133 labeling was localized in the plasma membrane (Fig. 1B). We then studied whether CD133 expression can be maintained for several days under hypoxic conditions. Cells were grown in 1% oxygen for 3 days, passaged twice and maintained in 1% oxygen for an additional 3 days. Flow cytometry analysis conducted daily during this time period revealed that 40–60% of U251MG cells became labeled with CD133 antibody. Moreover, when cells were returned in 21% oxygen (re-oxygenation), CD133 expression progressively decreased to normoxic levels over the next 3 days (Fig. 1C). These experiments demonstrate that hypoxic conditions increased the CD133 expression by the majority of U251MG glioma cells that normally express a low level of CD133, and that this event was reversible upon re-oxygenation. These findings have been reproduced using the D54MG glioma cell line (data not shown).

### Rotenone treatment enriched CD133 positive glioma cells

**Figure 2:**
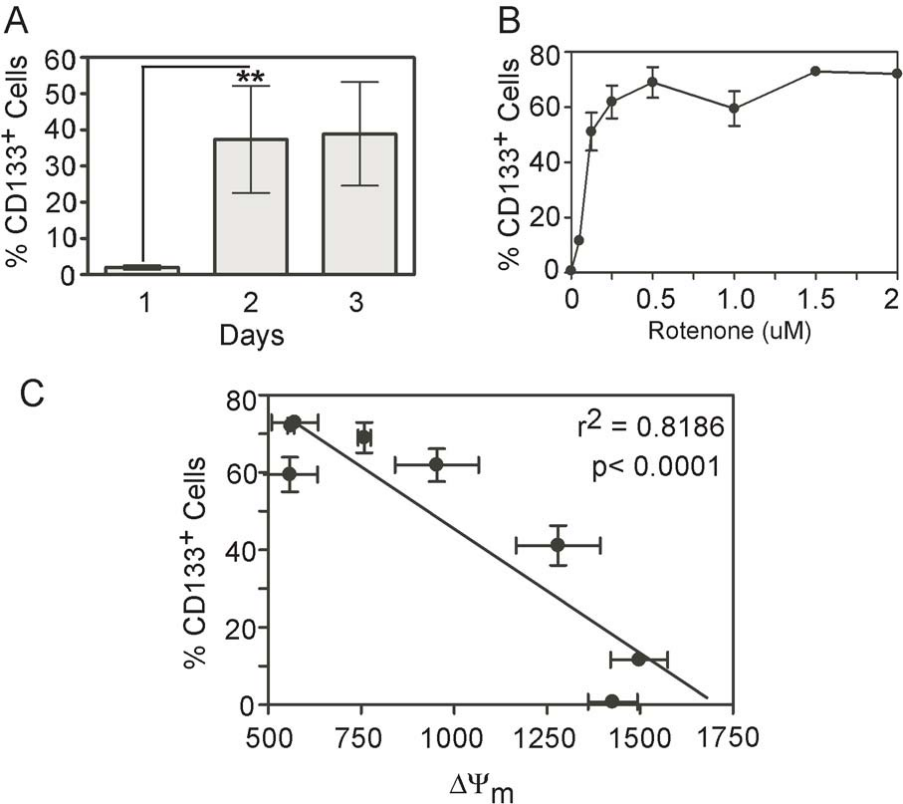
Rotenone enriched the population of CD133 positive glioma cells. A) Time-dependent expression of CD133 in U251 glioma cells exposed to 1 μM rotenone for up to 3 days. B) Dose-dependent regulation of CD133 positive cells. C) Relationship between percentage of CD133 positive cells and ΔΨm. Data were analyzed by linear regression and p value were calculated using Spearman correlation test. Results are given as means ± SD of 5 separate experiments. ** represent p<0.001.

In a recent study, we described that glycolytic glioma cells are resistant to blockers of the ETC, in particular rotenone, a complex I (NADH dehydrogenase) blocker [1], [2]. To determine whether CD133 positive glioma cells became enriched in presence of rotenone, we treated U251MG glioma cells with 1 μM rotenone for up to 3 days under normoxic conditions. Flow cytometric analysis indicated that 37±10 % of the cells became labeled with anti-CD133 antibody (Fig. 2A). Induction of CD133 by rotenone was dose-dependent (Fig. 2B). We analyzed the data by Spearman correlation test and by linear regression. Increases in the CD133 positive population after rotenone exposure were significantly correlated with decreases in their mitochondrial membrane potential (ΔΨm) (r_s_ = 0.82; p<0.0001) (Fig. 2C). These findings have been corroborated using the D54MG glioma cell line (data not shown).

### Expression of CD133 increases in mitochondrial DNA depleted human glioma cells

**Figure 3:**
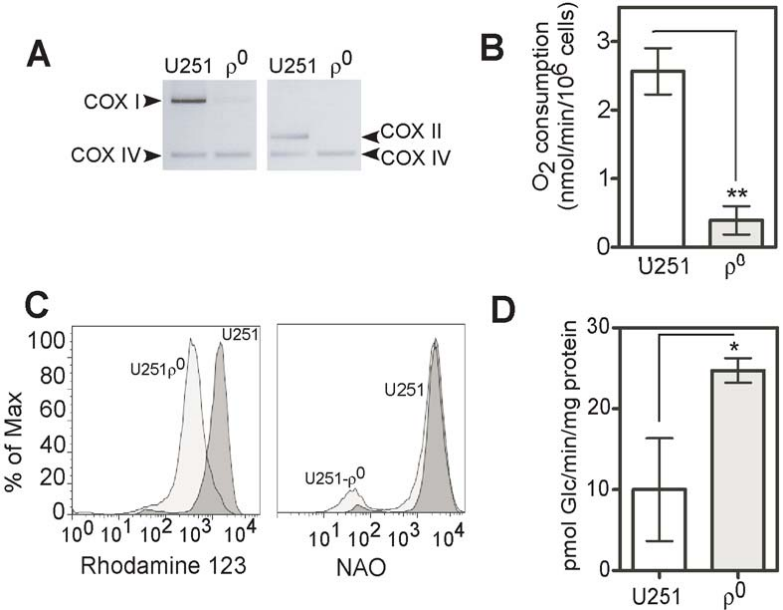
Bioenergetic characterization of mtDNA depleted U251 glioma cells. A) Genomic DNA was isolated from control and mtDNA-depleted U251 cells, and mtDNA-encoded genes including cytochrome c oxidase subunit I (COX-I) and cytochrome c oxidase subunit II (COX-II) were amplified by PCR. COX IV is used as a nuclear DNA-encoded control. The PCR products were electrophoresed on 1.5% agarose gel and then visualized by EtBr staining. A representative result of three independent experiments is shown. B) Average oxygen consumption rates in U251 and U251ρ^0^. Results are given as means ± SD of 5 separate experiments. C) Rhodamine123 fluorescence profiles of U251 and U251ρ^0^; NAO fluorescent profiles of U251 and U251ρ^0^. D) Glucose transport activity for the parental and ρ^0^ cells. Results are given as means ± SD of 3 separate experiments.

Numerous mtDNA-depleted cell lines (rho^0^, ρ^0^) have been generated by long-term treatment with very low doses of ethidium bromide [22], [23], [24] in order to study important mitochondrial defects in OxPhos, calcium homeostasis alteration, reactive oxygen species (ROS) production and resistance to apoptosis. In order to test whether loss of mitochondrial function by mtDNA depletion would also up-regulate appearance of CD133^+^ glioma cells, we generated U251MG mtDNA depleted cells, termed U251ρ^0^. The mtDNA content from U251MG cells cultured with or without EtBr was monitored routinely by amplifying total DNA. As shown in Figure 3A, cytochrome oxidase subunits I (COX I) and II (COX II) which are encoded only in mtDNA were barely amplified from the total DNA of the cells treated with EtBr. In contrast, nuclear DNA encoded COX IV was equally detected in both control and EtBr-treated cells, indicating that prolonged treatment with EtBr selectively depleted mtDNA without altering the nuclear DNA replication in U251MG cells.

After clonogenic selection under ethidium bromide, pyruvate, uridine and 72 serial passages, total oxygen consumption and glucose uptake were measured. Oxygen traces from a Clark electrode showed a time-dependent decrease in oxygen concentration for cell suspensions from ρ^0^ and parental glioma cells. However, ρ^0^ glioma cells consumed significantly less oxygen than the parental cell lines (p<0.005; student's *t* test) in nmol/min/10^6^ cells: 2.46±0.6 versus 0.4±0.5 for U251 versus U251ρ^0^ (Fig. 3B).

Mitochondrial membrane potential (ΔΨ_m_) and mitochondrial mass were assayed by flow cytometry. Decreased incorporation of Rhodamine 123 in ρ^0^ compared to parental cells indicated that mitochondrial function was indeed impaired. Total mitochondrial content, as revealed by cardiolipin detection using nonyl acridine orange, remained unchanged (Fig. 3C). These results indicated that ρ^0^ treatment perturbed mitochondrial function but did not change the quantity of mitochondria. Concomitantly, glucose uptake was significantly increased in U251ρ^0^ compared to U251 (p<0.05; student's t test), which we have shown as a glycolytic type [1], has elevated glucose transporters and glucose uptake (Fig. 3D).

**Figure 4:**
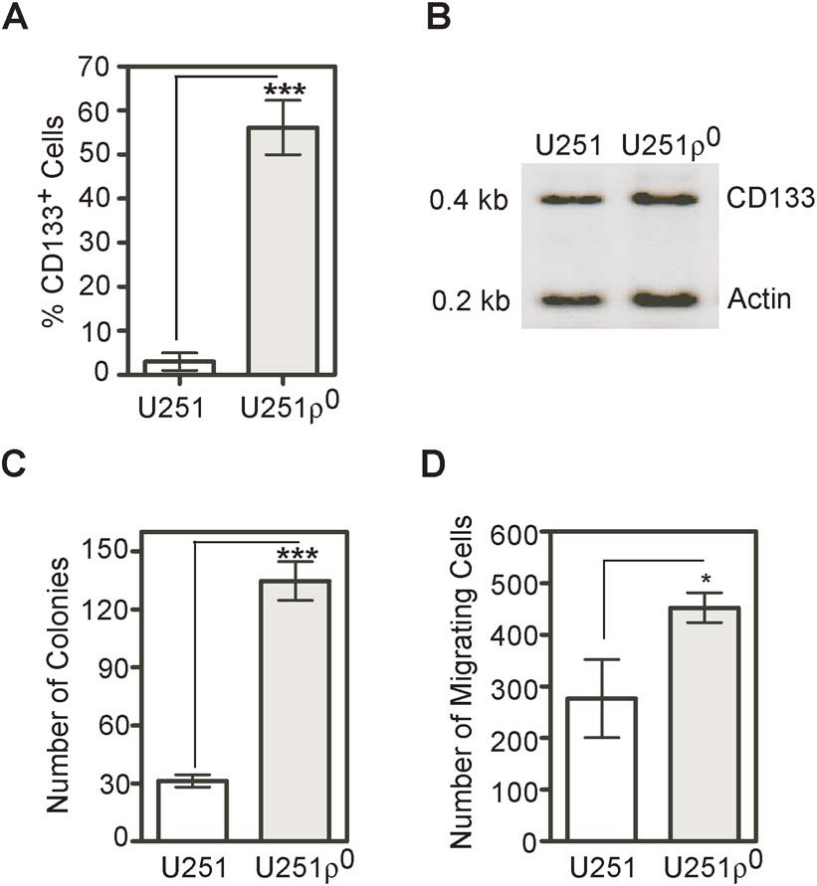
Anchorage-independent, invasion and CD133 expression in ρ^0^ glioma cells. A) Flow cytometry determination of the percentage of CD133 expressing parental and ρ^0^ cells. Results are given as means ± SD of 5 separate experiments. B) RT-PCR detection of CD133 mRNA from U251 and U251ρ^0^ glioma cells. Electrophoresis resolved amplicons for CD133 (0.4 kbp) and actin (0.2 kbp) of anticipated sizes. C) Colony formation in soft agar. Results are given as means ± SD of 3 separate experiments. D) Analysis of invasion into Matrigel 24 h after 5% FCS as chemo-attractant has been added to the lower chamber. *, **, *** represent p<0.05, p<0.005 and p<0.001.

To determine whether CD133 expression was up-regulated in ρ^0^ glioma cells, we analyzed CD133 expression by flow cytometry. Figure 4A illustrates that the CD133^+^ glioma cell population increased from 0.5±0.02% to 52.40±7.54% for U251ρ^0^ cells. This increased level of CD133 expression remained stable for up to 72 passages (data not shown). This finding raised the question of whether expression of CD133 was due to transcription or post-transcription mechanisms. RT-PCR revealed that CD133 mRNA was expressed in both parental and ρ^0^ glioma cells, suggesting that expression of CD133 messenger does not predict cell surface immunoreactivity with CD133 antibodies (Fig. 4B). It seems that expression of the CD133 protein is at least partly regulated at a post-transcriptional level. The relative levels of expression were consistent with that reported for CD133 transcripts in retinoblastoma, lung and colon adenocarcinoma [25].

In order to test the potential oncogenic transformation of mtDNA depleted cells, we performed anchorage-independent growth in soft agar and Matrigel invasion assays. Soft-agar plating efficiency was significantly increased in ρ^0^ cells compared to the parental glioma cells (p<0.001 for U251 versus U251ρ^0^; Fig. 4C; student's *t* test). Matrigel invasion assays showed that U251ρ^0^ are significantly more invasive than the parental U251 cells (p<0.05; student's *t* test) (Fig. 4D). Taken together; these data indicated that ρ^0^ glioma model depleted of their OxPhos pathways have a more aggressive transformed phenotype than their isogenic counterpart. Essentially, identical findings have been observed with the D54MG glioma cell line (data not shown).

### U251ρ^0^ glioma cells harbor properties ascribed to neural and brain tumor stem cells

Previous studies described that brain tumor stem cells (BTSCs), as well as their neural stem cell counterparts, are characterized by their ability to form neurospheres in defined serum-free culture medium supplemented with epidermal growth factor (EGF) and basic fibroblast growth factor (bFGF) [26]. Moreover, cells comprising neurospheres expressed nestin and prominin-1 (CD133), neural stem cell markers. Under differentiation conditions, BTSCs expressed markers of neuronal and glial lineages [7], [8], [9], [10], [11], [13], [27].

**Figure 5:**
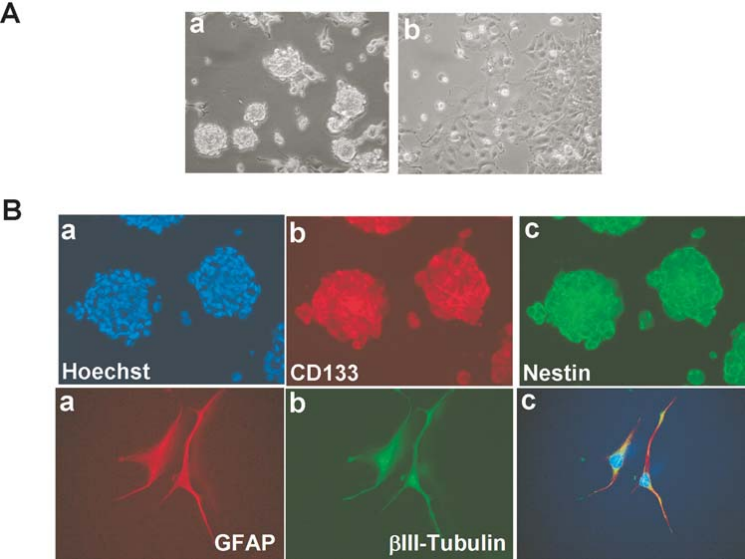
ρ^0^ glioma cells form neurosphere-like tumor spheroids expressing neural stem cell markers, nestin and CD133. A) Phase contrast microphotographs (magnification X20) of ρ^0^ glioma forming (a) neurosphere-like structures in serumless Neurobasal medium supplement with EGF and FGF after 10 days in culture or (b) adherent culture in DMEM/F12 medium with 7% FBS. B) Spheroids of ρ^0^ cells were immunostained with (b) CD133 or (c) Nestin antibodies or nuclear counterstained with Hoechst 33258 (a). C) Differentiation potential of ρ^0^ tumor spheroids. Multipotency was assayed by immunofluorescence for neuronal (βIII-tubulin) (a, c) and glial (GFAP) (b a, c) markers.

We used the culture conditions that favor neural stem cell growth, previously established for isolation of neural stem cells as neurospheres [26]. Neurobasal medium allows for the maintenance of an undifferentiated stem cell state, and the addition of bFGF and EGF induce proliferation of multipotent, self-renewing, and expandable neural stem cells [9], [10], [26]. Within 48–72 h, U251ρ^0^ cultures yielded cells that demonstrated growth into clonally-derived neurosphere-like clusters, which we termed tumor spheroids (Fig. 5A, a). Under DMEM/F12 supplemented with 7% fetal bovine serum, ρ^0^ U251 grows adherently to the plastic substrata and does not form tumor spheroids (Fig. 5A, b). Tumor spheroids retained expression of the classical neural stem cell markers CD133 and nestin (Fig. 5B, a-c, upper panels). We next employed conditions used for neurosphere differentiation to determine whether the U251ρ^0^ were capable of multilineage differentiation. After differentiation with 10% FBS for 7 days, immunocytochemistry was performed on U251ρ^0^ tumor spheroids using antibodies for neuron-specific β III-tubulin and, astrocyte-specific GFAP. As illustrated in Figure 5B, a-c, lower panels, cells from U251ρ^0^ tumor spheroids exhibited immunoreactivity for both markers indicating multilineage properties.

### Transmitochondrial cybrid cell lines shows parental phenotype

**Figure 6:**
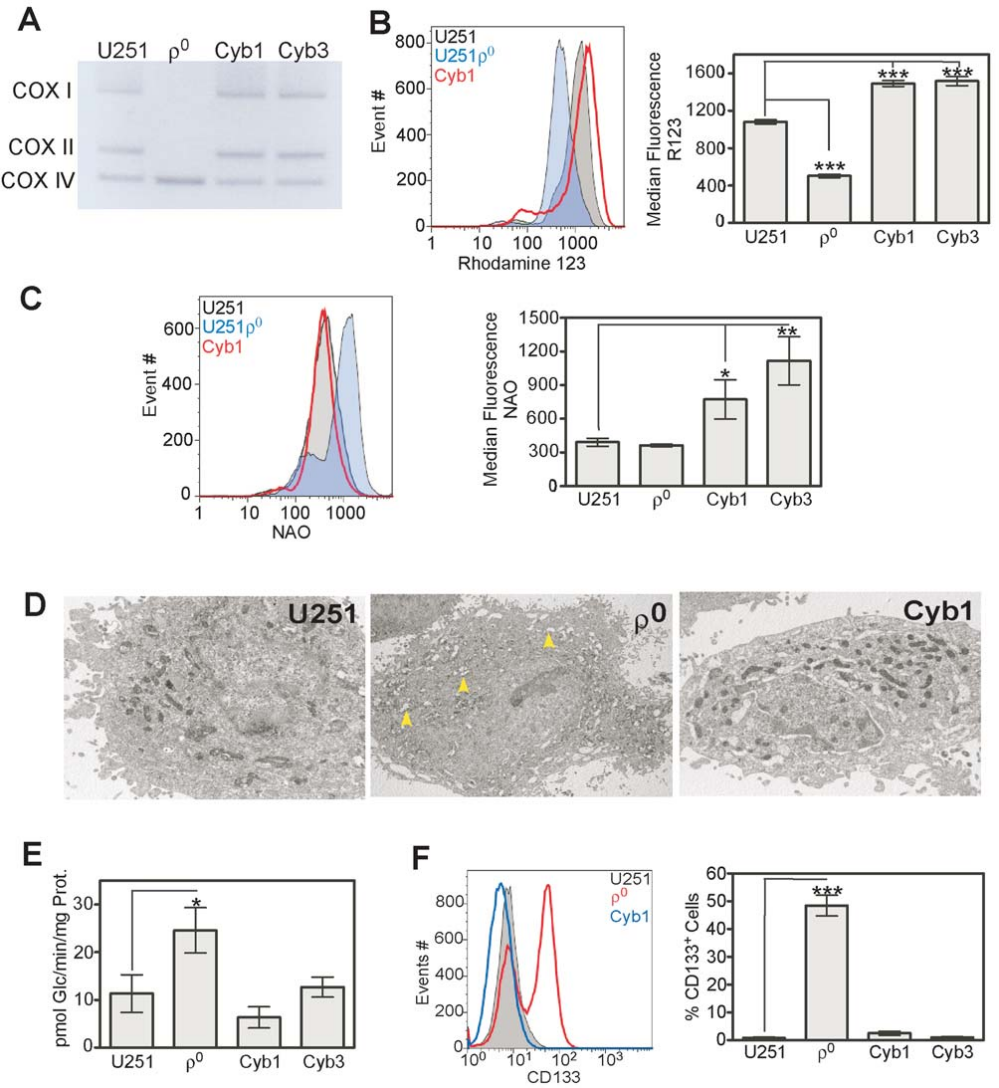
Characterization of U251 transmitochondrial cybrids. A) Genomic DNA was isolated from control, mtDNA-depleted and transmitochondrial cybrid cells, and mtDNA-encoded genes including COX-I and COX-II were amplified by PCR. COX IV serves as a nuclear DNA-encoded control. The PCR products were electrophoresed on 1.5% agarose gel and then visualized by EtBr staining. A representative result of three independent experiments is shown. B) Representative histograms of R123 fluorescence for U251, U251ρ^0^ and cybrids cells (left); average R123 fluorescence profiles (right). C) Representative histograms of NAO fluorescence for U251, U251ρ^0^ and cybrids cells (left); average NAO fluorescence profiles (right). D) A Representative electron transmission microscopy photographs comparing U251, U251ρ^0^ and cybrids cells (Magnification X6000). Arrowhead showing that U251ρ^0^ cells have smaller mitochondria with large intramembraneous spaces. E) Glucose transport activity for the parental and ρ^0^ cells. Results are given as means ± SD of 3 separate experiments. F) Representative histograms of flow cytometry CD133 fluorescence for U251, U251ρ^0^ and cybrids cells (left). Percentage of CD133 expressing cells (right). Results are given as means ± SD of 5 separate experiments.

In order to further demonstrate that up-regulation of CD133 is a consequence of mtDNA depletion, we repaired the defect by generating cybrid clones in which wild type parental (U251MG) mtDNA was transferred to ρ^0^ cells. Following the enucleation- fusion-selection protocol, six individual surviving cybrid clones were isolated. We selected 2 of these cybrid clones for subsequent analyses that by PCR showed repletion of the mitochondrial encoded cytochrome oxidases COX I and COX II to normal levels compared to U251MG cells. In contrast, nuclear-encoded mitochondrial COX IV remained unchanged in U251MG parental cells, in mtDNA depleted ρ^0^ cells or in the cybrid cells (Fig. 6A). These results indicated that ρ^0^ cells were repopulated with exogenous mtDNA from U251MG parental glioma cells resulting in transmitochondrial cybrids.

To determine Δψm after mtDNA repletion, we exposed living cells to rhodamine 123 which is preferentially taken up by mitochondria. Flow cytometric measurements revealed an increase in Δψm in both cybrid clones compared with ρ^0^ cells. Compared with U251MG, a 38±1.73% and 40±2.54% increase in average fluorescent intensity incorporated into mitochondria was detected in cybrid 1 and cybrid 3 respectively (*P*<0.005, Fig. 6B). Consonant results were also obtained by flow cytometry with NAO-stained U251MG, ρ^0^ and cybrid cells. The average fluorescence emission in ρ^0^ cells was not markedly different from that in parental U251MG cells. However, 1.97 and 2.84 fold-increases in NAO incorporation were detected in cybrid 1 and cybrid 3, respectively, compared with U251MG parental cells (*P*<0.05, Fig. 6C).

Figure 6D shows electron photomicrographs of parental U251MG, ρ^0^ and cybrid 1 cells. U251MG and cybrid 1 cells both contained homogeneous, elongated mitochondria. In contrast, most of the ρ^0^ cells visualized appeared to have swollen or enlarged mitochondria, showing either complete loss of cristae patterns with empty mitochondrial structure or a disorganized cristae pattern.

Energy production of ρ^0^ cells completely depends on anaerobic glycolysis and it stands to reason that glucose consumption of cybrid clones should be elevated. Reference values were obtained by examination of U251MG cells, which we have previously defined as largely, but not completely, glycolytic. Glucose consumption (Fig. 6E) of ρ^0^ cells (25±2.7 pmol Glc/min/mg protein), however, more than doubled the already increased rate seen in parental U251MG glioma cells (11±2.2 pmol Glc/min/mg protein). However, after repairing the mtDNA loss, glucose consumption by the cybrids decreased to values similar to that of U251MG cells (6±1.5 and 12±1.8 for cybrid 1 and cybrid 3 respectively). Additional evidence of the restoration of mtDNA is the fact that the cybrid clones no longer required supplementation with pyruvate and uridine for survival.

Cybrid cells derived from ρ^0^ cell lines were stained with fluorochrome-conjugated primary antibodies against CD133 and were analyzed by flow cytometry. FACS analyses indicated that CD133 was highly expressed in ρ^0^ cells but after repair of these mtDNA-depleted cells by fusion with U251MG cytoplasts, the elevation in CD133 expression was reversed to parental U251MG cell levels (Fig. 6F).

## Discussion

Hypoxia is a hallmark of glioblastoma multiforme tumors and is believed to promote cancer development, invasive dispersal of tumor cells, and resistance to therapy [28], [29], [30]. Because oxygen is the main substrate of the ETC, hypoxic conditions consequently decreases mitochondrial function and initiates expression of a host of genes that are needed for compensatory ATP production by glycolysis. We showed that glioma cells from long-term established cultures under ambient oxygen (21%) did not express CD133 until they were transferred to severe hypoxia (1% Oxygen) and then maintained their CD133 expression for as long as they were exposed to 1% O_2_. This CD133 expression on the cell surface was immediate, transient and completely reversible when the cells were returned to ambient oxygen conditions. It is well known that not all tumor cells undergo hypoxia at a fixed time during the formation of the tumor mass. Indeed, chaotic tumor angiogenesis may lead to heterogeneous blood perfusion (blood vessels regressing or collapsing). Thus, the supply of oxygen and/or glucose will likely fluctuate [6], [31], and expression of a “neural stem cell” marker such as CD133 could be transiently expressed as part of the “glycolytic response”.

It is possible that the low incidence of CD133-expressing cells observed in many studies is related to supraphysiologically high oxygen conditions of routine tissue culture. Indeed, we showed that under ambient oxygen less than 3% of U251MG cells expressed CD133. Similarly, low percentages have been described for D54MG [13] and U87MG [14] glioma cells. Two recent studies have described an enriched CD133-positive cell fraction in cells exposed to low oxygen concentration [27], [32]. Therefore, oxygen tension emerges as a main regulator of CD133 expression. Just as hypoxia plays a major role in tumor development, a similar trend is observed during embryogenesis [33], [34], [35]. Indeed, hypoxia is known to maintain human embryonic stem cells (ES) in undifferentiated phenotypes [20]. Upon return to normoxia, ES begin to differentiate.

Thus, hypoxia, leading to deprivation of the main electron acceptor, causes perturbation of mitochondrial membrane potential (Δψ) and decreases the coupling efficiency between oxidation and phosphorylation. We previously demonstrated that the presence in the culture medium of rotenone, an inhibitor of the respiratory chain Complex I, significantly decreased Δψ, without affecting cell viability in U251MG glioma cells [2]. We have shown here that rotenone caused a significant increase in CD133 expression by glioma cells, and this effect was significantly correlated with the inhibition of the mitochondrial membrane potential, demonstrating that mitochondrial function plays a crucial role in the regulation of CD133 expression. However, a definitive and specific role of Complex I in CD133 regulation remains to be elucidated.

The importance of mitochondria in tumor biology has its roots in Warburg's original studies [4], [36]. He postulated that the origin of cancer was due to an irreversible damage of mitochondrial function, leading to enhanced glycolysis. Many studies since then have confirmed increased glycolytic capacity in many malignant tissues, including glioma. However, other studies have demonstrated that cancer cells have functional mitochondria [37], [38]. Our previous study identified several glioma cell lines that were dependent on mitochondrial OxPhos pathway while others were largely dependent on glycolysis. Critically important, glycolytic glioma cells were able to switch energy production to OxPhos in order to survive under limiting glucose conditions, indicating that dual bioenergetic capabilities [1], [39], [40] can be revealed using the appropriate stressors. Thus, mitochondria of glycolytic glioma are functional and essential for survival under low glucose conditions.

We initiated this study to investigate the possible link between mitochondria function and tumor progression in human glioma. *In vitro*, mammalian cells can be depleted of their mitochondrial DNA, creating ρ^0^ cells [22], [24], [41]. ρ^0^ cells lack a functional electron transport chain and appear incapable of generating ATP via the OxPhos mitochondrial pathway. We generated ρ^0^ glioma models that are characterized by decreased O_2_ consumption, decreased mitochondrial membrane potential and increased glucose uptake. These cells are auxotrophic for uridine and pyruvate and grow quite well without a functional ETC. We showed that ρ^0^ glioma cells developed enhanced anchorage-independent growth and increased invasion phenotypes. These enhanced tumorigenic properties of mtDNA-depleted glioma cells is in accordance with studies reported in mtDNA-depleted human pulmonary carcinoma cells and HeLa cells [12], [42]. To our knowledge, this is the first report of a ρ^0^ model in human glioma. One key feature of this model is the stable expression of CD133. Indeed, the proportion of CD133-expressing cells remains constant during more than 72 serial passages. Moreover, our mtDNA-depleted glioma cells, when grown under defined serumless medium, were able to form tumor spheroids that closely resembled neurospheres that develop under identical conditions from normal neural stem cells. Moreover, mtDNA-depleted glioma cells displayed the capacity for long-term proliferation, self- renewal, and multipotency. All of these properties are classical features of neural stem cells [26] or brain tumor stem cells [7]. The defects in respiratory function following induction of the mtDNA depletion in ρ^0^ glioma cells were reversed by repletion of mtDNA with from enucleated U251MG cytoplasts. Restoration of mitochondrial function in cybrid cells resulted in down-regulation of CD133 expression to parental cell levels. This finding indicates that mitochondrial encoded proteins and their associated functions in the cells play a role in the expression of CD133.

**Figure 7:**
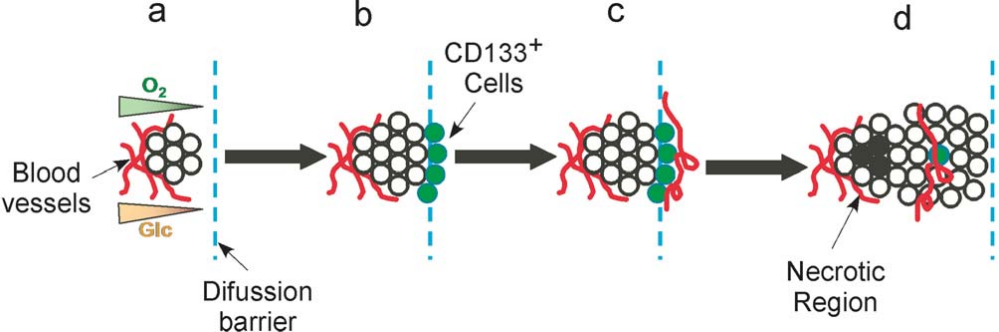
Tumor progression model: (a) Initiation of tumor formation. (b) Tumor growth restricted at the oxygen and glucose diffusion barrier. (c) Glioma ells at the edge of the tumor will be re-oxygenated by neovascularization, and grow and divide until they reach a new oxygen and nutrient diffusion barrier. (d): repetitive cycles of growth and angiogenesis occur during the progression of the tumor.

Our data support a revised model of tumor progression, where epigenetic factors play a critical determinant role [43]. In this tumor progression model, the origin of the first transformed cell is irrelevant but focuses on adaptive biological processes induced in tumor cells through genetic or mitochondrial mutations [44]. This model places a stronger emphasis on the effect of the microenvironment, which is universally recognized has one of the main driving forces of tumor progression [6], [45], [46] (Fig. 7). Our model considers the following factors: (a): nascent tumor cells begin to divide until they have produced nutrient and oxygen diffusion barriers [6]. These cells encounter stringent growth limitation through lack of oxygen and nutrients. A significant fraction of these cells that are close to the most stringent hypoxic microenvironment will have a significant loss of mitochondrial OxPhos function and subsequently switch to glycolysis. This glycolytic switch will trigger expression of stem cell markers such as CD133 and multiple phenotypic changes that result in increased invasion, migration and angiogenesis. (b): increased angiogenesis will result in formation of new vessels that will provide a transient increase in the microenvironment content of oxygen and nutrients allowing the switch from glycolysis back to OxPhos metabolism. (c): because the cells at the margin of tumor invasion will be re-oxygenated, they will grow and divide generating a new oxygen and nutrient diffusion barrier. (d): many such cycles of growth and angiogenesis are repeated during the progression of the tumor. In this model, a feature of the bioenergetically stressed phenotype of glioma cells, as defined by the microenvironment, is expression of CD133. Thus, the number of CD133-positive cells fluctuates, adding a dynamic and transitory dimension to the progression of the tumor.

Another feature of this microenvironment driven phenotype accommodation that validates this model, is the capacity of glioma cells to switch bioenergetic pathways as has been demonstrated by our group and others [1], [2], [40], [47], [48]. Glioma cells that constitute the bulk of the progressively growing tumor need be extremely plastic, resistant to cell death in unfavorable growth conditions, but most at all, opportunistic of their environment, as has been suggested [49]. Glioma cells with this capability represent a functionally unique subpopulation of cancer cells that manifest “cancer stem cell” properties. The difficulty in designing more efficient therapies lies in understanding how these subpopulations of cells respond to these microenvironmental challenges by engaging different survival pathways.

A critically important observation from our study is that metabolic flexibility is necessary and essential for tumor progression in glioma. Induction of two opposing phenotypes in cells of identical genetic background demonstrates the plasticity, multipotency and opportunistic properties of these tumors. It suggests that malignant glioma cells are capable of adapting to and remodeling their microenvironment, switching phenotypic behavior to improve their chances for survival. We understand the outcome of becoming more invasive to move to a more hospitable microenvironment, but the expression and function of classic stem cell markers, nestin and CD133, as survival attributes is less clear.

We described here that hypoxia and modification of the bioenergetic status of glioma cells govern the regulation of CD133 at post-transcriptional level. Data presented here strongly indicated that changes in the cellular environment that results in alteration of mitochondrial function are responsible for the enhanced up-regulation of CD133 antigen in glioma cells, suggesting that CD133 expression in human glioma cells is not obligatory relative to the stem cell phenotype but rather, reveals the occurrence of a stress response.

## Materials and Methods

### Cell and culture and hypoxia treatment

Human glioma cell lines U251-MG and D54-MG were originally obtained from Dr. D.D. Bigner (Duke University, Durham, NC). Parental U251-MG and D54-MG cells were cultured using 50:50 mixture of DMEM and Ham's nutrient mixture F12 (MediaTech) supplemented with 7% heat- inactivated fetal bovine serum (HyClone) and 2.6 mmol/L L-glutamine. Cells were treated with *in-vitro* hypoxia for up to 7 days at 1% O_2_ in a hypoxia chamber (Coy, Laboratory). For re-oxygenation experiments, cells were returned to 21 % O_2_ for the designated period of time. All chemicals were purchased from Sigma Chemical Co. (St. Louis, MO), unless otherwise stated.

### Staining of mitochondria

Glioma cells were plated in 12-well plates and cultured for 24 h. Cell monolayers were harvested using trypsin-EDTA (Gibco), washed with Dulbecco's PBS and stained with mitochondrial fluorescent dyes 10-N-nonyl-Acridin Orange (NAO, 1 μM) or Rhodamine 123 (R123, 5 μM) for flow cytometric analysis. After 15 min at 37°C, samples were washed once with 2% FCS/PBS to remove unbound dyes, resuspended in 2% FCS/PBS with 2 μg/ml of propidium iodide to allow exclusion of dead cells by FACS. Flow cytometry was performed using a FACSCalibur (Becton Dickinson) and analyzed with FlowJo software (Tree Star, Inc.). Each analysis included at least 20,000 events.

### Flow cytometric analysis for CD133

Glioma cells were incubated in cell staining buffer containing biotin-conjugated anti-AC133/1 antibody (Miltenyi Biotec) followed by incubation with Streptavidin-APC-Cy5.5. Isotype-matched immunoglobulin served as controls. Cells were incubated for 20 min at each step and were washed with 2% FCS/PBS between steps. After staining, cells were resuspended in 2% FCS/PBS with 2 μg/ml of propidium iodide to identify dead cells. Two-color flow cytometric analyses were performed using a FACScalibur (Becton Dickinson) and FlowJo software (Tree Star, Inc). Each analysis included at least 25,000 events. The percentage of CD133^+^ cells present was assessed after correction for the percentage of cells reactive with an isotype control.

### Immunocytochemical staining of CD133 glioma cells

Cells were cultured on 12 mm poly-lysine coated glass cover slides. After treatment, cells were incubated in staining buffer containing biotin-conjugated anti-AC133/1 antibody (Miltenyi Biotec) followed by incubation with Alexa Fluor 555 streptavidin. Cells were incubated for 20 min at each step and were washed with 2% FCS/PBS between steps. After staining cells were fixed with 4% paraformaldehyde (15 min, room temp) and rinsed 3 times with D-PBS. Cells were nuclear counterstained with 4′6-diamidino-2-phyenylindole (DAPI), washed and mounted in Fluormount G (Southern Biotechnology Associates) and viewed with a Leica/Leitz DMRB fluorescence microscope equipped with appropriate excitation and emission filter sets (Chromatechnology). Images were acquired with a C5810 series digital color camera (Hamamatsu Photonic System). Images were processed with Adobe Photo Shop and IP LAB Spectrum software (Signal Analytics Software).

### Reverse Transcription-Polymerase Chain Reaction Analysis of CD133

Total RNA was extracted using RNeasy Micro Kit (Qiagen). Reverse transcriptase polymerase chain reaction (RT-PCR) was performed on cDNA using primers specific for CD133 and actin as previously described [38]. PCR products were run on 2% agarose gel with ethidium bromide and visualized using the Eagle Eye Gel reader (Stratagene).

### Generation of mtDNA depleted glioma cells (ρ^0^ cells)

U251-MG and D54-MG cells were cultured in 50 ng/ml Ethidium bromide in DMEM/F12 supplemented with 7% FBS, 1 mmol/L pyruvate and 50 μg/ml uridine. Cells were seeded at low density onto 6 well plates and individual colonies propagated and screened for uridine and pyruvate auxotrophy. Cells were passage twice a week and medium changed every other day.

### Oxygen Consumption Measurements

Cells from triplicate cultures were trypsinized and suspended at 1×10^6^ cells/ml in DMEM/ F12 supplemented with 7% FBS. Oxygen consumption was measured in a 1-ml volume gas-tight vessel thermostatted at 37°C, equipped with a Clark type oxygen electrode (Hansatech Instrument Ltd) as described [50]. The data were exported to a computerized chart recorder (Oxygraph 1.01, Hansatech, Instrument Ltd), which calculated the rate of oxygen consumption expressed as nmol oxygen consumed/min/10^6^ cells. Carbonyl cyanide methoxy phenylhydrasone (FCCP), a mitochondrial uncoupler was used to assay maximal oxygen consumption rate.

### ^14^C-2-deoxy-D-glucose (DOG) uptake

^14^[C]-DOG was purchased from Amersham Biosciences (Piscataway, NJ) (61 mCi/mmol). Uptake experiments were carried out as we have described previously [1].

### Anchorage independent clonogenic assay

Glioma cells (2,000) previously mixed with 0.45% agar-DMEM/F12 supplemented with 7% fetal bovine serum were seeded on a 0.9% agar cushion. Cells were fed every 5 days with DMEM/F12. After 3 weeks, the colonies were fixed with PBS-formalin, stained with Giemsa and counted.

### *In vitro* invasion assay

Cell invasion assays were conducted in BD BioCoat matrigel invasion transwell chambers with 8.0-μm pore membranes (BD Biosciences). Cells (4×10^4^) in 500 μL culture medium were added to the upper chamber. The bottom chambers contained 800 μL culture medium supplemented with 7% FCS as a chemoattractant. At 24 h later, the upper membranes were wiped with cotton swabs and any cells that had migrated to the bottom side of the membranes were fixed in ice-cold methanol. Hematoxylin and eosin staining was performed and membranes were excised, placed on microscope slides. Cells in five random visual fields (100× magnification) were counted for each membrane. All experiments were done in triplicates.

### Transmitochondrial cybrid generation

To obtain transmitochondrial cybrids, human U251MG glioma cells were enucleated as previously described [51], [52]. Briefly, cells were treated with cytochalasin B (10 μg/ml) and then layered on a Ficoll step gradient. After centrifugation at 37°C for 90 minutes at 130,000 g cytoplasmic fractions were collected. The cytoplasts were then fused with U251 ρ^0^ cells by adding a solution of 50% polyethylene glycol (MW 1450, Sigma cat P-5402) in phosphate buffer. Cybrids were plated and continuously grown in DMEM/F12 medium supplemented with 7% dialyzed FBS. Two weeks after fusion, cybrids were plated at low density for clonogenic selection.

### Transmission electron microscopy

Electron photomicrographs for parental U251MG, U251 ρ0 and transmitochondrial cybrid cells were prepared as described previously [53]. Briefly, cell pellets were washed in sucrose/phosphate buffer and fixed in paraldehyde and glutaraldehyde. Pellets were then post-fixed in phosphate-buffered 1% osmium tetroxide. Samples were stained en bloc with 2% uranyl acetate, rinsed, dehydrated, and embedded in Spurr's resin. Thin sections were cut on an Ultracut E ultramicrotome (Reichert-Jung), placed on copper grids, stained with lead citrate, and micrographed with an EM-1200 EX II electron microscope.
